# Large Language Models in Hematology Case Solving: A Comparative Study of ChatGPT-3.5, Google Bard, and Microsoft Bing

**DOI:** 10.7759/cureus.43861

**Published:** 2023-08-21

**Authors:** Amita Kumari, Anita Kumari, Amita Singh, Sanjeet K Singh, Ayesha Juhi, Anup Kumar D Dhanvijay, Mohammed Jaffer Pinjar, Himel Mondal

**Affiliations:** 1 Physiology, All India Institute of Medical Sciences, Deoghar, Deoghar, IND; 2 Pathology, All India Institute of Medical Sciences, Deoghar, Deoghar, IND

**Keywords:** ai and robotics in healthcare, microsoft bing, google bard, chatgpt, pathology, hematology, hematologic diseases, natural language processing, search engine, pathologists

## Abstract

Background

Large language models (LLMs), such as ChatGPT-3.5, Google Bard, and Microsoft Bing, have shown promising capabilities in various natural language processing (NLP) tasks. However, their performance and accuracy in solving domain-specific questions, particularly in the field of hematology, have not been extensively investigated.

Objective

This study aimed to explore the capability of LLMs, namely, ChatGPT-3.5, Google Bard, and Microsoft Bing (Precise), in solving hematology-related cases and comparing their performance.

Methods

This was a cross-sectional study conducted in the Department of Physiology and Pathology, All India Institute of Medical Sciences, Deoghar, Jharkhand, India. We curated a set of 50 cases on hematology covering a range of topics and complexities. The dataset included queries related to blood disorders, hematologic malignancies, laboratory test parameters, calculations, and treatment options. Each case and related question was prepared with a set of correct answers to compare with. We utilized ChatGPT-3.5, Google Bard Experiment, and Microsoft Bing (Precise) for question-answering tasks. The answers were checked by two physiologists and one pathologist. They rated the answers on a rating scale from one to five. The average score of the three models was compared by Friedman’s test with Dunn’s post-hoc test. The performance of the LLMs was compared with a median of 2.5 by a one-sample median test as the curriculum from which the questions were curated has a 50% pass grade.

Results

The scores among the three LLMs were significantly different (p-value < 0.0001) with the highest score by ChatGPT (3.15±1.19), followed by Bard (2.23±1.17) and Bing (1.98±1.01). The score of ChatGPT was significantly higher than 50% (p-value = 0.0004), Bard's score was close to 50% (p-value = 0.38), and Bing's score was significantly lower than the pass score (p-value = 0.0015).

Conclusion

The LLMs reveal significant differences in solving case vignettes in hematology. ChatGPT exhibited the highest score, followed by Google Bard and Microsoft Bing. The observed performance trends suggest that ChatGPT holds promising potential in the medical domain. However, none of the models was capable of answering all questions accurately. Further research and optimization of language models can offer valuable contributions to healthcare and medical education applications.

## Introduction

Hematology, the specialized field of medicine focused on the study of blood and its associated disorders, holds paramount importance in the realm of healthcare. Precise diagnosis and effective management of hematologic conditions, such as anemia, leukemia, and coagulopathies, are imperative for optimizing patient outcomes and improving overall public health [1].

The advent of artificial intelligence (AI) and natural language processing (NLP) has led to the development of large language models (LLMs), which exhibit exceptional capabilities in processing and comprehending natural language data [2]. Prominent among these LLMs are ChatGPT, Google Bard, and Microsoft Bing, which have garnered substantial interest due to their capacity to comprehend textual information and generate contextually relevant responses [3]. These models have demonstrated various levels of accuracy in performing medical examinations, solving complex medical issues, or interpreting radiology reports [4,5,6,7,8].

Nevertheless, their efficacy and suitability for domain-specific applications, particularly in addressing medical inquiries pertaining to hematology, remain relatively unexplored. The intricate nature of hematology, characterized by a lexicon replete with specialized terminology and a wide array of conditions with nuanced diagnostic and therapeutic considerations, necessitates meticulous scrutiny of language models' performance in this context.

The present study aimed to address this gap in knowledge by exploring the capability of LLMs in solving hematology cases and conducting a comparative analysis of three LLMs, namely, ChatGPT, Google Bard, and Microsoft Bing.

## Materials and methods

Study type and settings

This was a cross-sectional study conducted in the Department of Physiology and Pathology, All India Institute of Medical Sciences, Deoghar, Jharkhand, India. The data were collected from three LLMs, namely, Google Bard Experiment (https://bard.google.com/), Microsoft Bing Precise Conversation based on GPT-4 (https://www.bing.com/), and ChatGPT-3.5 Free Research version (https://chat.openai.com/), available free to any registered users. We used the free version of the LLMs. Henceforth in the article, the models are mentioned as Bard, Bing, and ChatGPT.

Hematology cases

**Figure 1:**
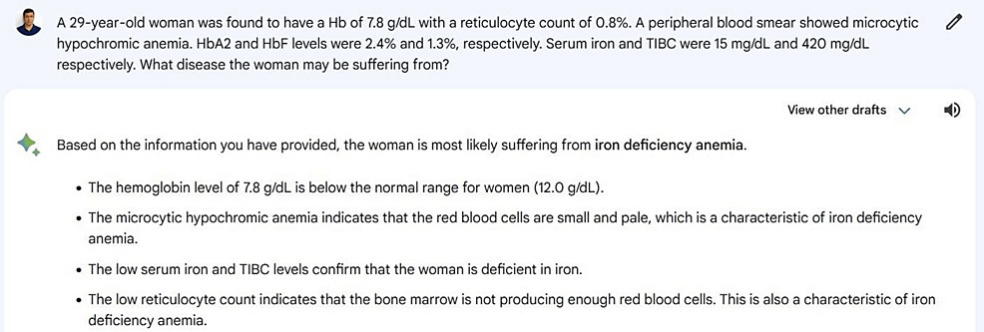
An example of a question and a part of the answer generated from Google Bard Screenshot captured from Google Bard (https://bard.google.com). The image in the screenshot is that of the corresponding author.

A set of hematology-related cases covering various topics and complexities were carefully designed by subject matter experts. These questions encompassed laboratory calculations, disease interpretations, and other relevant aspects of hematology. A total of 50 cases and related questions were made, and content validity was checked by two experts of curriculum design. An example question and a part of the answer generated from Google Bard are shown in Figure 1.

**Figure 2:**
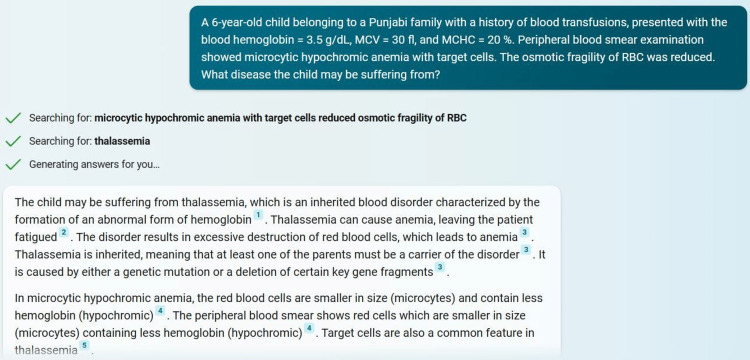
An example of a question and a part of the answer generated from Microsoft Bing Chat Screenshot captured from Microsoft Bing (https://www.bing.com/).

Another example question and a part of the answer generated from Microsoft Bing are shown in Figure 2.

**Figure 3:**
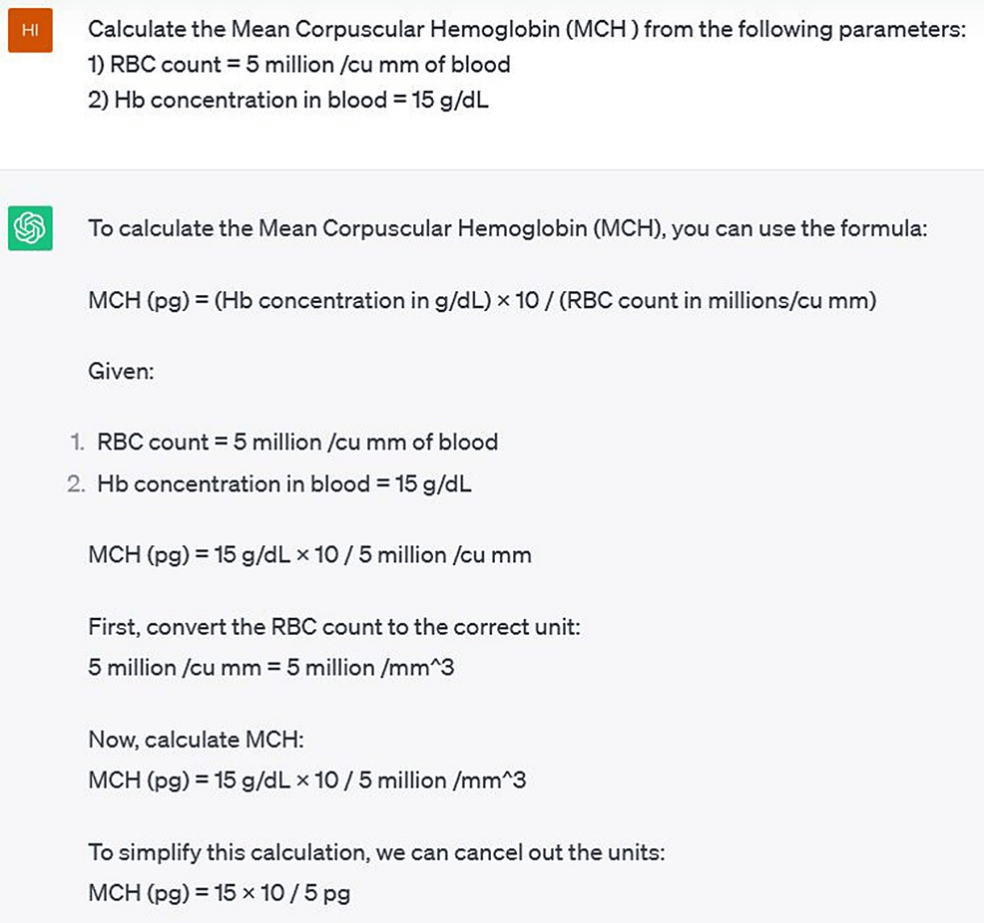
An example of a question and a part of the answer generated from ChatGPT-3.5 Screenshot captured from ChatGPT (https://chat.openai.com/).

Another example is shown in Figure 3, where the response was generated from ChatGPT-3.5.

Data collection from LLMs

The questions were asked to three LLMs, namely, Bard, Bing, and ChatGPT, on July 30, 2023 to get answers. Generated answers were stored for further analysis. The answers were coded and blinded to the raters to reduce bias.

Assessing accuracy by three raters

To assess the accuracy of the LLMs' responses, three independent raters, with expertise in hematology and medical education, were recruited. The raters evaluated each LLM-generated answer and scored them based on their correctness with an accuracy score ranging from 1 to 5. The detailed scoring method was as follows:

5 - Highly accurate: The answer provided by the AI is thoroughly accurate, aligning perfectly with clinical knowledge and best practices. 4 - Moderately accurate: The answer provided by the AI is mostly accurate, with only minor discrepancies that do not significantly impact its clinical reliability. 3 - Somewhat accurate: The answer provided by the AI contains several inaccuracies that may require clarification or verification by a medical professional. 2 - Slightly accurate: The answer provided by the AI has noticeable inaccuracies, and its clinical reliability is questionable without substantial correction. 1 - Inaccurate: The answer provided by the AI is fundamentally incorrect and could pose serious risks to patient care if relied upon without thorough review and correction.

Data analysis

The obtained raters' evaluations were tested for normality by the Shapiro-Wilk test. The data were found not to follow normal distributions. Hence, we used nonparametric tests. The data were presented in mean, standard deviation (SD), median, first quartile (Q1), and third quartile (Q3) [9]. The average (average of the three raters) score awarded to the three LLMs was compared by Friedman’s test with Dunn’s post-hoc analysis. The average score was also compared by a one-sample Wilcoxon signed-rank test with a hypothetical value of 2.5 (50% score is the passing score in the curriculum of hematology from where the cases and questions were framed). Intraclass correlation coefficient (ICC) was calculated to determine the agreement level among the raters in assessing the accuracy of the LLMs' responses [10]. We used IBM SPSS Statistics for Windows, version 20 (released 2011; IBM Corp., Armonk, New York, United States) for statistical analysis. A p-value < 0.05 was considered statistically significant.

## Results

A total of 50 cases were analyzed by the three raters. The scores among the three LLMs were significantly different (p-value < 0.0001), with the highest score by ChatGPT (3.15±1.19), followed by Bard (2.23±1.17) and Bing (1.98±1.01).

**Figure 4:**
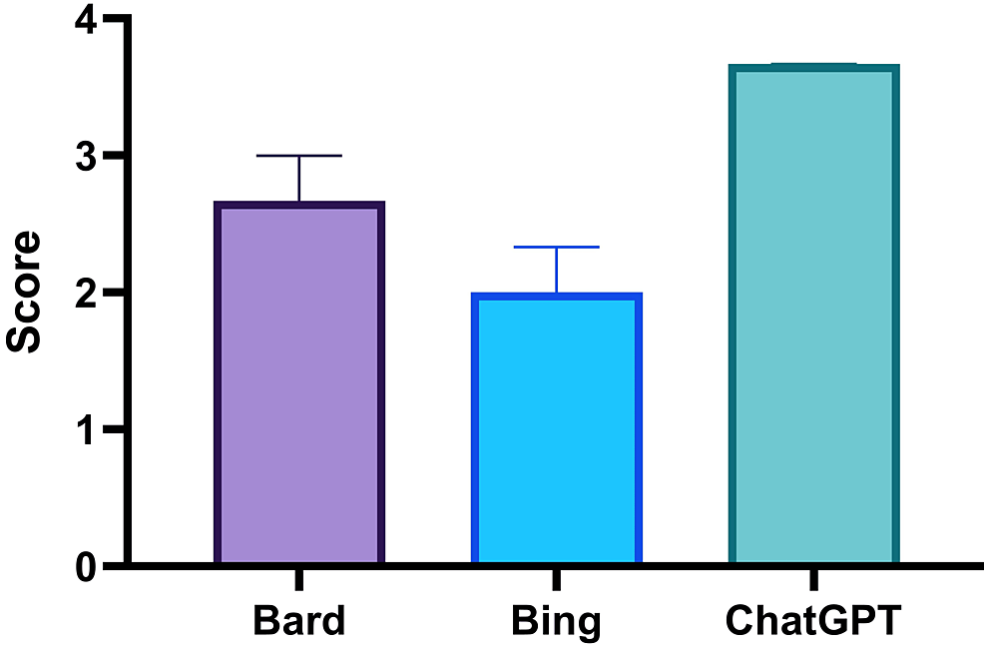
Average scores of Bard, Bing, and ChatGPT in the answers to the hematology questions The bar and whisker indicate a median score with a 95% confidence interval. The comparison was done by Friedman’s test (non-parametric analysis of variance (ANOVA)) with Dunn’s post-hoc analysis. The median and first quartiles/third quartiles were as follows: Bard 2.67 (1.67/3), Bing 2 (1.33/2.67), and ChatGPT 3.67 (3/4).

The median score with 95% confidence interval (CI) is shown in Figure 4. In the post-hoc analysis, Bard versus Bing did not show any significant difference (p-value = 0.33). However, Bard versus ChatGPT (p-value < 0.0001) and Bing versus ChatGPT (p-value < 0.0001) showed significant differences.

The scores of the three raters are shown in Table 1. The scores among the raters showed a good to excellent level of reliability.

**Table 1:** Scores of the answers by the three LLMs as rated by the three raters *Statistically significant p-value of the intraclass correlation coefficient (ICC) [10] LLM: large language model, SD: standard deviation Interpretation of ICC: <0.5 = poor reliability, 0.5-0.75 = moderate reliability, 0.75-0.9 = good reliability, >0.90 = excellent reliability [9]

LLM	Central tendency	Rater 1	Rater 2	Rater 3	ICC, p-value
Google Bard	Mean±SD	1.88±1.19	2.12±1.41	1.94±1.11	0.83, <0.0001*
	Median (Q1-Q3)	3 (1-4)	2 (0.25 - 3)	2 (1-3)	
Microsoft Bing	Mean±SD	2.52±1.42	2.02±1.41	2.16±1.18	0.78, <0.0001*
	Median (Q1-Q3)	2 (1-3)	2 (1-3)	2 (1-3)	
ChatGPT-3.5	Mean±SD	3.4±1.26	3.02±1.25	3.02±1.27	0.98, <0.0001*
	Median (Q1-Q3)	4 (3.2-4)	3 (3-4)	3 (3-4)	

When we conducted the Wilcoxon signed-rank test with a hypothetical value of 2.5 (the course from where the questions were prepared needs a 50% score to pass), we found that ChatGPT’s score was significantly higher than the passing score (p-value = 0.0004). The score of Bard was close to 50% (p-value = 0.38). However, Bing’s score was significantly lower than the passing score (p-value = 0.0015).

## Discussion

We found that ChatGPT performed better than the other two LLMs in problem-solving abilities in hematology. These findings emphasize the importance of selecting the most appropriate language model based on its performance for specific tasks, enabling more effective problem-solving in various scenarios. The LLMs are evolving day by day. Hence, further studies are required in the future to fully explore their capability [11]. When we tested the models' performance with a minimum passing score (50%) of the curriculum, we found ChatGPT to pass it with the highest margin, indicating that it consistently outperformed the threshold. Meanwhile, Bard's score showed no significant difference from the passing score, suggesting that it performed at a level close to the passing requirement. By contrast, Bing's score was significantly lower than the passing score, indicating that it fell short of meeting the passing standard. These results highlight the varying proficiency levels of the language models in achieving accuracy with the same questions at the same time.

Based on the finding that laboratory calculations or interpretations for hematology diseases are weak in many cases, caution should be exercised when considering the use of LLMs in medical education. While LLMs can provide valuable information and learning resources, their limitations in accurately handling laboratory data for hematology diseases warrant careful supervision and validation by qualified medical educators. In the context of healthcare, where precision and accuracy are crucial, the use of LLMs for making clinical decisions or providing direct patient care should be approached with caution. LLMs can serve as helpful tools for information retrieval and initial insights, but they should not replace the expertise and clinical judgment of healthcare professionals. As technology advances and LLMs undergo further refinement, they may hold more promise for enhancing medical education and supporting healthcare, but their current limitations necessitate prudent utilization. Some of the potential use of LLMs are summarized in Table 2 [12,13,14,15,16].

**Table 2:** Potential audience and brief where large language models (LLMs) can help

Category	Brief
Students	LLMs can act as virtual tutors, helping medical students access supplementary information, explanations, and case studies to reinforce their learning.
Healthcare professionals	Doctors, nurses, and other healthcare professionals can use LLMs to stay updated with the latest medical research, treatment guidelines, and evidence-based practices.
Patients	LLMs can be used in health applications or chatbots to provide basic medical information and answer common health-related queries for patients.
Researchers	LLMs can assist researchers in searching for relevant literature, summarizing papers, and extracting key insights from a vast amount of medical literature.
Remote health assistants	LLMs can be particularly valuable in areas with limited access to healthcare resources, where individuals can seek initial medical advice and information.

The substantial agreement among the raters may be attributed to several potential reasons. The raters were provided with clear and well-defined evaluation criteria, ensuring a consistent understanding of the scoring method. Their familiarity and expertise in assessing hematology-related questions could have contributed to more aligned judgments. However, in-depth analysis and examination of the specific data and rater feedback would be necessary to fully understand the underlying factors influencing the agreement among the raters.

Limitations

The study has several limitations that should be considered when interpreting the findings. The sample size of hematology questions and LLM responses was relatively small, potentially impacting the generalizability of the results. Rater bias and subjectivity in evaluating LLM responses might have introduced variability. Only three models were tested. The cross-sectional design offers only a snapshot of LLM performance, and real-world applications may present additional challenges. Despite these limitations, this study provides valuable insights into LLM accuracy in hematology questions and underscores the need for further research and exploration of ethical considerations in utilizing LLMs in medical education and healthcare.

## Conclusions

There were variations in the LLM performance, with ChatGPT demonstrating the highest accuracy, Bard exhibiting moderate accuracy, and Bing showing comparatively lower accuracy in answering questions of hematology. While LLMs hold promise as valuable tools for medical education, caution is warranted in their use, considering their limitations in handling complex medical nuances and potential inaccuracies. This study emphasizes the need for continuous refinement and validation of LLMs for reliable healthcare applications.
